# A Randomized Study to Compare a Monthly to a Daily Administration of Vitamin D_3_ Supplementation

**DOI:** 10.3390/nu10060659

**Published:** 2018-05-23

**Authors:** Sophie De Niet, Monte Coffiner, Stéphanie Da Silva, Bernard Jandrain, Jean-Claude Souberbielle, Etienne Cavalier

**Affiliations:** 1 Clinical Department, Laboratoires SMB SA, 1080 Brussels, Belgium; sdeni@smb.be (S.D.N.); mcoff@smb.be (M.C.); 2 Department of Clinical Pharmacology, ATC SA, 4000 Liège, Belgium; bernard.jandrain@atc-pharma.be; 3 Laboratoire d’Explorations fonctionnelles, Hôpital Necker-Enfants malades, Paris 75014, France; jean-claude.souberbielle@nck.aphp.fr; 4 Department of Clinical Chemistry, University of Liège, CHU Sart-Tilman, 4000 Liège, Belgium; Etienne.cavalier@chu.ulg.ac.be

**Keywords:** vitamin D, calcidiol, calcitriol, FGF23, regimen, supplementation

## Abstract

We aimed to determine whether a cumulative dose of vitamin D_3_ produces the same effects on the serum concentration of 25(OH)D_3_ if it is given daily or monthly. This is a monocentric, two-armed, randomized, interventional, open, and parallel study conducted from November 2016 to March 2017 in Belgium. We randomized 60 subjects with vitamin D deficiency to receive 2000 IU vitamin D_3_ daily or 50,000 IU monthly. The same cumulative dose of vitamin D_3_ was given to each treatment group (150,000 IU). The 25(OH)D_3_ serum concentrations from baseline to day 75 were 14.3 ± 3.7 to 27.8 ± 3.9 ng/mL in the monthly group and 14.1 ± 3.4 to 28.8 ± 5.4 ng/mL in the daily group. The mean change versus the baseline level was significantly different between the groups at day 2, 4, 7, and 14 and no longer different from day 25. One day after the intake of vitamin D_3_, as expected, serum 25(OH)D_3_ and 1,25(OH)_2_D_3_ increased significantly in the monthly group, whereas they did not change significantly in the daily group. The median time to reach the 20 ng/mL target concentration was significantly different in the two groups, in favor of the monthly regimen (1 day versus 14 days; *p* = 0.02). In conclusion, a monthly administration of 50,000 IU vitamin D_3_ provides an effective tool for a rapid normalization of 25(OH)D_3_ in deficient subjects. A daily administration of the same cumulative dose is similarly effective but takes two weeks longer to reach the desirable level of 20 ng/mL.

## 1. Introduction

A low vitamin D status is common worldwide and may affect health in terms of its effects on bone, fracture, muscle strength, and fall risks. The known functions of vitamin D have grown to include roles in immune function, cardiovascular health, and cancer prevention. Vitamin D insufficiency has been recognized in a large proportion of the general population, with higher deficiencies among women, elderly, and minorities. Compared with the general population, vitamin D deficiency is more common in patients with hyperparathyroidism and is often associated with an aggravated form of the disease. The problem is even more relevant in patients with chronic kidney disease (CKD), where vitamin D deficiency and the resultant secondary hyperparathyroidism have been associated with an increase of cardiovascular morbidity and mortality, mainly due to vascular and soft tissue calcifications [1,2,3,4,5]. Vitamin D is activated into 25(OH)D_3_ (calcidiol) in the liver and then converted into 1,25(OH)_2_D_3_ (calcitriol), the active form of vitamin D, in the kidney. Current guidelines from scientific bodies around the globe recommend the measurement of 25(OH)D_3_ in the blood as the preferred test for the assessment of vitamin D status. This recommendation is based on numerous studies that have demonstrated significant associations of 25(OH)D_3_ with biochemical, functional, and clinical indices, such as parathyroid hormone (PTH), neuromuscular function, bone mineral density, and fracture risk [6,7,8,9]. The essential question of how much vitamin D is needed for optimal bone and global health remains unsolved. According to different guidelines, the thresholds for serum 25(OH)D_3_ are set at 20 or 30 ng/mL (i.e., 50 or 75 nmol/L) for bone health. The guidelines focused on pleiotropic effects of vitamin D recommend a target 25(OH)D concentration of 30 ng/mL [10]. Most guidelines recommend 600 IU or 800 IU per day to reach 25(OH)D_3_ serum concentrations of 20 ng/mL. The Endocrine Society guidelines recommend a higher intake (up to 2000 IU per day or higher until the target level is reached) for a long list of individuals at increased risk of vitamin D deficiency [11,12,13,14,15,16]. Treatment compliance is another key factor in the management of bone health, as in other chronic diseases [17]. The main point is related to the fact that, in clinical practice, maintaining long-term adherence to daily dosages of vitamin D is often difficult, especially in pediatric and elderly populations [18,19]. Long-term compliance with treatment has been shown to be poor in osteoporosis patients, and about half of the patients stop the therapy within one to two years [20]. Since the pharmacology of vitamin D indicates a half-life suitable to dose intervals longer than daily, more convenient dosing regimens with high vitamin D doses at less frequent intervals have become widespread practice [21]. Until now, there are no consistent data suggesting the ideal regimen of supplementation, and the question of the ideal time between doses is still of debate. Ish-Shalom et al. [22] performed a study in elderly women to compare the efficacy and safety of a daily dose of 1500 IU to a weekly dose of 10,500 IU and to a dose of 45,000 IU given every 28 days for two months. They concluded that supplementation with vitamin D can be equally achieved with daily, weekly, or monthly dosing frequencies. Another study comparing daily, weekly, and monthly supplementation of vitamin D in deficient patient was published by Takacs et al. [23]. They reported an equal efficacy of 1000 IU taken daily, 7000 IU taken weekly, and 30,000 IU taken monthly. Nevertheless, these consistent findings differ from the report by Chel et al. [24] in which a daily dose was more effective than a monthly dose. It is interesting to note that, in that study, the compliance calculation could be questionable, as only random samples of the returned medications were counted. 

Since the studies published so far show conflicting results concerning the daily and monthly supplementation of vitamin D, the objective of the present study was to determine whether a same cumulative dose of vitamin D_3_ produces different effects on the serum concentration of 25(OH)D_3_ and 1,25(OH)_2_D_3_ if it is given daily or monthly .

## 2. Materials and Methods

### 2.1. Methodology

This was an interventional, randomized, two-treatments, two-arms, open, and parallel study which was carried out in one clinical site in Belgium. The study was performed in accordance with the ethical principles that have their origin in the Declaration of Helsinki and that are consistent with Good Clinical Practice and the requirements according to the National Drug Law. All subjects provided written informed consent, and the study was approved by an Independent Ethics Committee (CHU, Liège, Belgium) and by the Belgian Competent Authorities (EudraCT No. 2016-003755-29).

**Figure 1:**
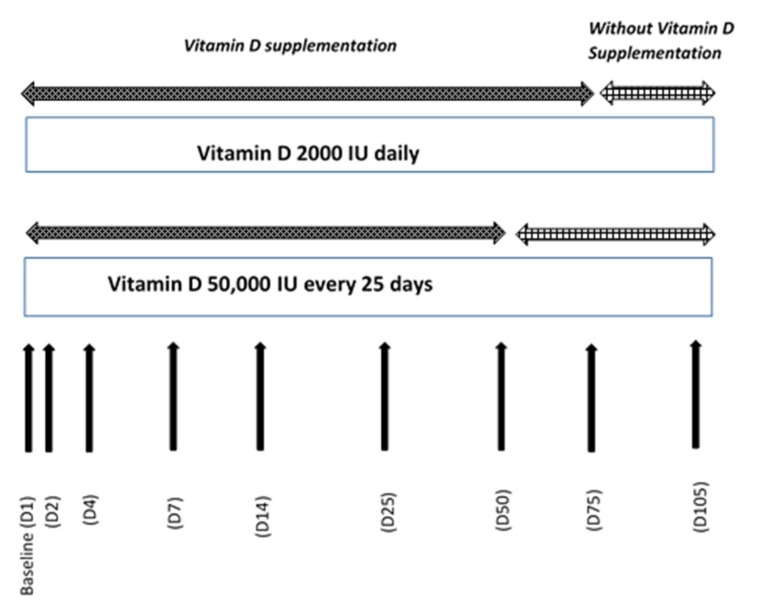
Study design.

Sixty subjects of both sexes were enrolled in the study in November 2016. They were randomized in two different regimen treatment groups and received the same total dose of vitamin D_3_ supplementation over a period of 75 days followed by a period without supplementation until Day 105. The two groups of subjects were treated in parallel. They received: one tablet for oral use containing 2000 IU of vitamin D_3_ per day from day 1 to day 75 (VISTA-D3^®^-Life, Pharma, Belgium) or two ampoules with an oily solution of vitamin D_3_ for oral use, containing 25,000 IU on day 1, day 25, and day 50 (D-CURE^®^-SMB TECHNOLOGY, Marche-en-Famenne, Belgium) in order to obtain the same cumulative dose after 75 days (Total dose: 150,000 IU). The compliance was checked by collecting all used and unused medications. Blood samples were collected at screening and before vitamin D_3_ supplementation on days 1, 2, 4, 7, 14, 25, 50, 75, and 105. The study ended for all subjects in March 2017. The study design is summarized in Figure 1.

### 2.2. Study Population

Caucasian healthy subjects, male and female, aged from 18 to 55 years, with vitamin D deficiency (defined as serum 25(OH)D_3_ concentration between ≥10 ng/mL and ≤20 ng/mL), and a body mass index (BMI) between 18 and 25 kg/m^2^ inclusive at screening were recruited.

The main exclusion criteria were any unstable clinically significant immunological, neoplastic, endocrine, hematological, hepatic, cardiac, renal, gastrointestinal, neurological or psychiatric abnormalities, or medical disease, past or current granulomatosis, sarcoidosis, urinary lithiasis, renal insufficiency, osteomalacia, abnormal digestive functions and abnormal thyroid function. Subjects who had a serum creatinine >150 µmol/L and an albumin-corrected serum calcium >2.65 mmol/L were excluded at screening. Finally, subjects who had used a UV light solarium or any type of vitamin D supplement within two months before the screening visit or planned to travel outside Europe during the study were excluded. Subjects were not allowed to take concomitant medications that were expected to interfere with the interpretation of the study data.

### 2.3. Laboratory Tests

At each study visit, blood samples were collected to determine the serum concentration of biomarkers: 25(OH)D_3_ was measured with the LC–MS/MS Vitamin D Standardization Program (VDSP)-certified method that has been described previously [25], whereas 1,25(OH)_2_D_3_, third-generation Parathyroid hormone (PTH), and intact Fibroblast growth factor (FGF23) were measured by the DiaSorin Liaison XL analyzer. Albumin and total calcium were measured by the Roche Cobas instrument. Corrected calcium was then calculated. At screening and at the end of the study routine, chemical assays were performed for safety purposes, including testing for phosphate, alkaline phosphatase, and Isotope dilution mass spectrometry (IDMS)-traceable creatinine (Roche Cobas). A blood pregnancy test was done for all females of childbearing potential. All the analyses were performed at the ISO 15198 clinical chemistry laboratory of the University of Liège (Liège, Belgium).

### 2.4. Statistical Analyses

The primary endpoint was the mean change in 25(OH)D_3_ serum level from baseline (D1 predose) to D75, which was compared between treatment groups by a mixed model, considering group, baseline value, and group*baseline as fixed factors and subjects as random factors. Changes were described with the estimated difference between the means and the corresponding two-sided 95% confidence interval. In addition, a paired *t*-test was performed within groups to test if the change between baseline and end-point was significant. The serum levels of 25(OH)D_3_, 1,25(OH)_2_D_3_, PTH, and FGF23 at each time point were compared by a mixed model for repeated measures with subjects as random factors. Multiple pairwise comparisons were conducted if the group*time interaction was significant. The area under the curve of 25(OH)D_3_ level (baseline to D75) was calculated using the trapezoidal methods. It was compared between the two groups, using a t test for independent samples. All statistical computations were performed using the SAS/STAT software version 9.4. (SAS Institute Inc., Cary, NC, USA) of the SAS system for Windows.

## 3. Results

All randomized subjects (60) completed the study. Their demographic data were similar in both groups at baseline (*p* > 0.05). The data are shown in Table 1.

The main efficacy analysis was conducted on the full analysis set. The analysis performed on the per-protocol population confirmed the results. The compliance during the study was 100% in the monthly regimen group and 99.6 ± 1.1% in the daily group.

### 3.1. Evolution of 25(OH)D_3_

Serum concentrations in 25(OH)D_3_ were similar in both groups at baseline (*p* = 0.86).

Not surprisingly, in the monthly group, after the first intake of 50,000 IU of vitamin D_3_, the serum concentration of 25(OH)D_3_ rapidly increased reaching mean values above 20 ng/mL as early as the day after the intake. After the second and third intake of 50,000 IU of vitamin D_3_, 25 and 50 days later, respectively, the concentration of 25(OH)D_3_ continued to increase slightly. At Day 105, despite the last intake of vitamin D_3_ was at day 50, the serum concentration of 25(OH)D_3_ was decreased but remained above 20 ng/mL. With daily administration of 2000 IU of vitamin D_3_, the increase in 25(OH)D_3_ serum concentration was less rapid at the beginning of the supplementation and reached a similar concentration to the monthly supplementation at Day 25. Once the daily supplementation was stopped at Day 75, the serum concentration of 25(OH)D_3_ decreased, remaining above 20 ng/mL four weeks later (Day 105), similarly to the monthly supplementation.

**Figure 2:**
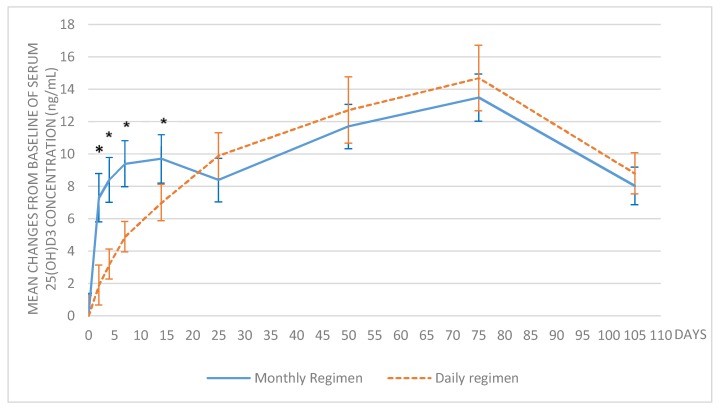
Mean change of 25(OH)D_3_ serum concentrations over time. The error bars show the 95% confidence interval; * *p* < 0.05 between the treatment groups.

In the monthly regimen group, the mean 25(OH)D_3_ serum concentration was 14.3 ± 3.7 ng/mL at baseline and 27.8 ± 3.9 ng/mL at day 75, with a mean change from baseline of 13.5 ± 5.5 ng/mL (*p* < 0.0001). In the daily regimen treatment group, the mean 25(OH)D_3_ serum concentration was 14.1 ± 3.4 ng/mL at baseline and 28.8 ± 5.4 ng/mL at day 75, with a mean change from baseline of 14.7 ± 7.0 ng/mL (*p* < 0.0001). The mean changes from baseline were significantly different between the treatment groups at day 2, 4, 7, and 14 (*p* < 0.05) because of the expected rapid increase after the monthly supplementation, and no longer different from day 25 (*p* > 0.05) (Figure 2).

To compare the extent of absorption after 75 days of supplementation, the area under the curve (AUC) was calculated from baseline to day 75. The AUC D1–D75 of 25(OH)D_3_ was 1837.6 ± 228 ng/mL*day in the monthly group and 1817.8 ± 288 ng/mL*day in the daily group, without significant difference between the two groups (*p* = 0.77), confirming similar effects independent of the treatment regimen.

### 3.2. Evolution of 1,25(OH)_2_D_3_

**Figure 3:**
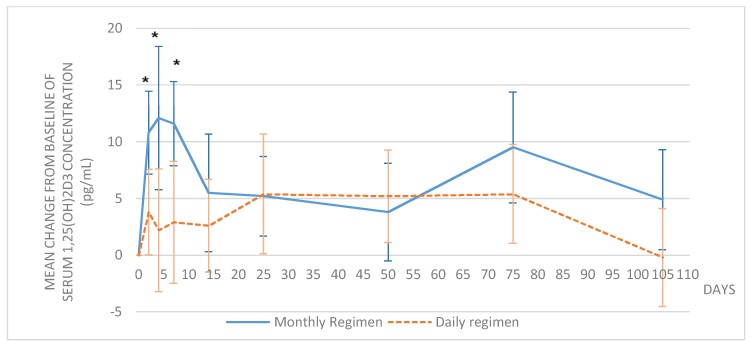
Mean change of 1,25(OH)_2_D_3_ serum concentrations over time. The error bars show the 95% confidence interval; * *p* < 0.05 between treatment groups.

The serum concentrations of 1,25(OH)_2_D_3_ were similar in both groups at baseline (*p* = 0.10). In the monthly group, the increase in 1,25(OH)_2_D_3_ versus baseline was significant as early as Day 2 and remained significantly higher than baseline until day 75 (*p* = 0.0004) and later until day 105 (*p* = 0.03), despite the last intake of vitamin D_3_ was at day 50. In the daily group, the increase from baseline was statistically significant after 25 days (*p* = 0.04) of vitamin D_3_ supplementation but returned to the baseline value at day 105 (*p* = 0.91). The mean changes from baseline were significantly different between the treatment groups at day 2, 4, and 7 (*p* < 0.05), and no longer different from day 14 (Figure 3).

### 3.3. Time to Definite Achievement of 25(OH)D_3_ Target

In the monthly group, the median time to reach the target of 20 ng/mL of 25(OH)D_3_ serum concentration was one day [1.0; 3.0]. In the daily group, the median time to reach the target was 14.0 days [7.0; 24.0] (*p* = 0.02 between groups).

### 3.4. Safety Assessment

There were no treatment-related adverse event (AE) and no drop-out reported during the study. No significant difference was found in reported laboratory parameters (albumin, creatinine, phosphates, alkaline phosphatase, and corrected calcium) among the treatment groups (Table 2). A trend toward a PTH decrease was observed in both groups, being significant in the monthly group only from D14 (−4.21 ± 1.45 ng/ml, *p* < 0.03). Globally, the serum FGF23 concentrations remained stable during the study in both groups, except at D75, when, in the daily group only, there was a significant increase (6.1 ± 15.9 pg/mL, *p* = 0.04).The maximum serum level of 25(OH)D_3_ observed was 35 ng/mL in the monthly group and 42 ng/mL in the daily group at day 75.

## 4. Discussion

In the present study, daily and monthly administrations of vitamin D_3_ in equivalent cumulative dosage have been evaluated in order to determine whether an intermittent therapy generates the same vitamin D_3_ status in a deficient population. The supplementation with a daily or monthly 2000 IU equivalent daily dose of vitamin D_3_ was shown to be effective in the restoration of 25(OH)D_3_ values above 20 ng/mL after 75 days of treatment, as 100% of the deficient subjects reached that cut-off. The mean levels failed to attain the 30 ng/mL threshold even after three months of supplementation, suggesting that higher doses may be required for adequate vitamin D repletion. These results are consistent with previously reported observations. In our study, we closely monitored the evolution of vitamin D levels over time and, not surprisingly, the monthly supplementation produced a more rapid increase in 25(OH)D_3_ and 1,25(OH)_2_D_3_ serum concentrations. The cut-off value of 20 ng/mL in 25(OH)D_3_ was reached 24h after the intake of vitamin D_3_, while 14 days were needed after the daily supplementation. The higher dose given in the monthly regimen group did not raise any safety concerns. There was no difference in the frequency of adverse events and other safety parameters between daily and monthly administrations. No treatment-related adverse event was reported, and no change in serum calcium concentration was observed. The monthly dose of 50,000 IU was proven equally safe as the 2000 IU daily dose in a vitamin D-deficient population. The same dose was evaluated by Binkley et al. [26] during a period of one year. Supplementation with 50,000 IU taken monthly by adult patients did not raise any safety concern. The safety of higher doses has been evaluated in multiple trials without any concerns, even if very largely spaced doses are not recommended, as shown in a study of Sanders et al. where a single annual dose of 500,000 IU resulted in an increased risk of falls and fractures [27,28,29,30,31]. No consensus has been reached about the dosages causing toxicity or about the upper safe limit of levels of 25(OH)D_3_. In our study, the maximum serum level of 25(OH)D_3_ observed was 35 ng/mL in the monthly group and 42 ng/mL in the daily group at day 75.

Our study has certain limitations. First, because of the exclusion criteria, our safety data do not reflect all aspects of real life. Second, the study was not blinded, although blinding was not necessary because the serum 25(OH)D_3_ could not have been influenced by a placebo effect. Even if the study was performed during the winter period to reduce UVB exposure as much as possible, differences between groups could be possible. The study was performed with non-lactating women, and the results cannot be applicable to breastfeeding women. The main strengths of our study include the prospective, randomized, controlled design. All used and unused treatments were checked in order to assess the compliance of the subject to the treatment. The compliance was checked regularly and was above 99.5% for both treatments. The same daily equivalent vitamin D_3_ was taken in order to obtain exactly the same cumulative dose after 75 days. Pharmacokinetic assessment was done regularly during the first days after administering the dose to characterize the short-term effect of a higher dose. The recruitment of subjects was performed controlling the range of BMI values. Another advantage of the study is the reliable and well-validated method used to measure the 25(OH)D_3_ concentrations in serum, using an assay calibrated according to the Vitamin D Standardization Program.

## 5. Conclusions

In conclusion, our results show that a monthly dose of 50,000 IU of vitamin D_3_ rapidly and safely normalizes 25(OH)D_3_ levels in deficient subjects. Daily 2000 IU dosing is similarly effective and safe. Nevertheless, the median time to reach the 20 ng/mL target concentration is significantly different between the two groups, in favor of the monthly regimen (1 day versus 14 days; *p* = 0.02). A monthly administration of 50,000 IU vitamin D_3_ does not raise safety concerns and provides an effective tool for the normalization of 25(OH)D_3_ to the desirable level of 20 ng/mL in deficient patients.

## Figures and Tables

**Table 1:** Demographic data of the 60 subjects included in the study.

		Monthly Regimen *n* = 30	Daily Regimen *n* = 30
Age (years)			
	Mean ± SD	29.8 ± 9.5	29.4 ± 7.7
	[min–max]	[19.0–53.0]	[19.0–44.0]
Gender			
Male	*N* (%)	9 (30%)	13 (43.33%)
Female	*N* (%)	21 (70%)	17 (56.67%)
BMI (kg/m^2^)	Mean ± SD	22.0 ± 2.1	21.9 ± 1.9
	[min–max]	[18.2–25.0]	[18.3–25.0]

BMI: body mass index; SD: standard deviation.

**Table 2:** Blood concentrations of safety parameters over time.

	Daily Regimen (Mean ± SD)	Monthly Regimen (Mean ± SD)
**ALBUMIN (g/L)**		
Baseline	46.7 ± 2.5	46.9 ± 2.3
Day 105	46.6 ± 2.4	46.1 ± 2.4
Change (Day 105—Baseline) *	−0.1 ± 2.3	−0.8 ± 2.3
**CREATININE (mg/dL)**		
Baseline	0.8 ± 0.2	0.8 ± 0.1
Day 105	0.8 ± 0.2	0.9 ± 0.1
Change (Day 105—Baseline) *	−0.0 ± 0.1	0.0 ± 0.1
**PHOSPHATES (mmol/L)**		
Baseline	1.0 ± 0.2	1.1 ± 0.2
Day 105	1.0 ± 0.2	1.1 ± 0.2
Change (Day 105—Baseline) *	0.0 ± 0.1	0.0 ± 0.2
**ALKALINE PHOSPHATASE (IU/L)**		
Baseline	57.0 ± 17.5	58.7 ± 16.2
Day 105	57.6 ± 17.7	58.4 ± 16.9
Change (Day 105—Baseline) *	0.6 ± 7.6	−0.3 ± 5.0
**CORRECTED CALCIUM (mmol/L)**		
Baseline	2.2 ± 0.1	2.2 ± 0.1
Day 105	2.2 ± 0.1	2.2 ± 0.1
Change (Day 105—Baseline) *	−0.0 ± 0.1	−0.0 ± 0.1
**PTH (ng/L)**		
Baseline	18.2 ± 6.0	22.3 ± 10.7
Day 105	16.5 ± 6.1	20.0 ± 9.0
Change (Day 105—Baseline) *	−1.6 ± 4.8	−2.3 ± 9.1
**FGF23 (pg/mL)**		
Baseline	51.9 ± 16.4	57.3 ± 16.3
Day 105	63.0 ± 18.8	63.6 ± 14.3
Change (Day 105—Baseline) *	11.1 ± 14.8	6.3 ± 15.9

* *p* > 0.05 between treatment groups.
